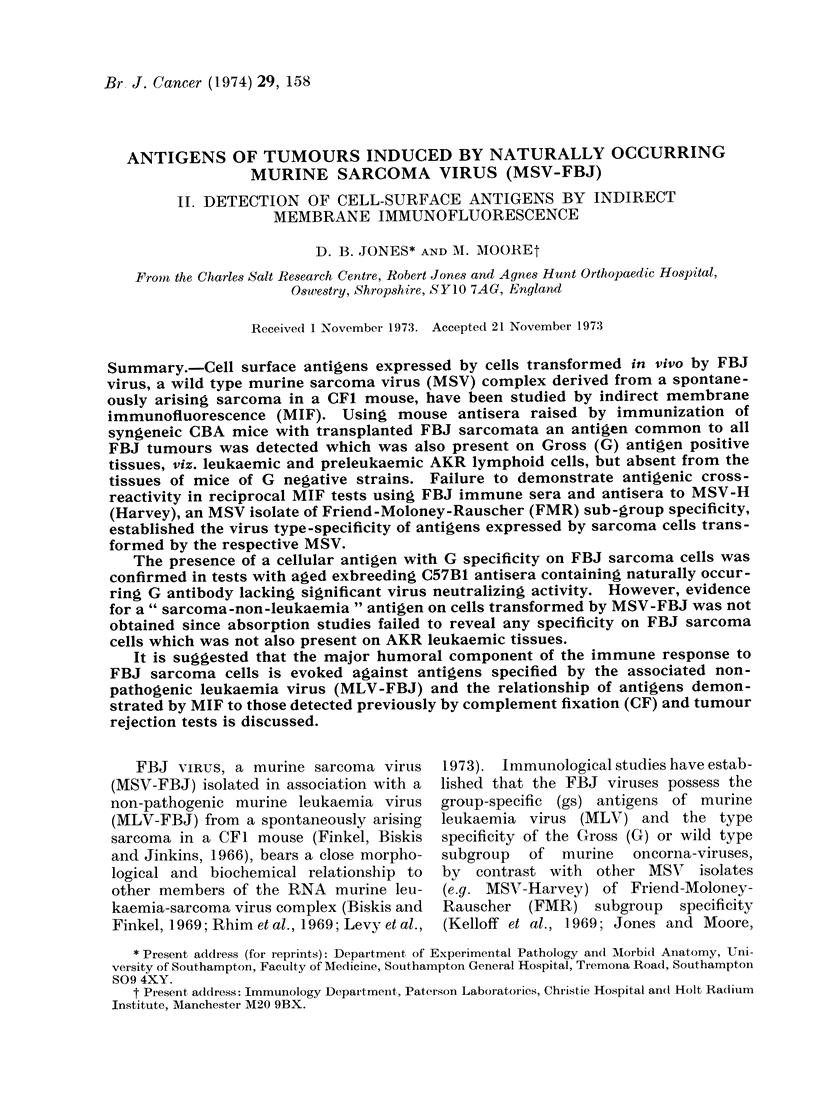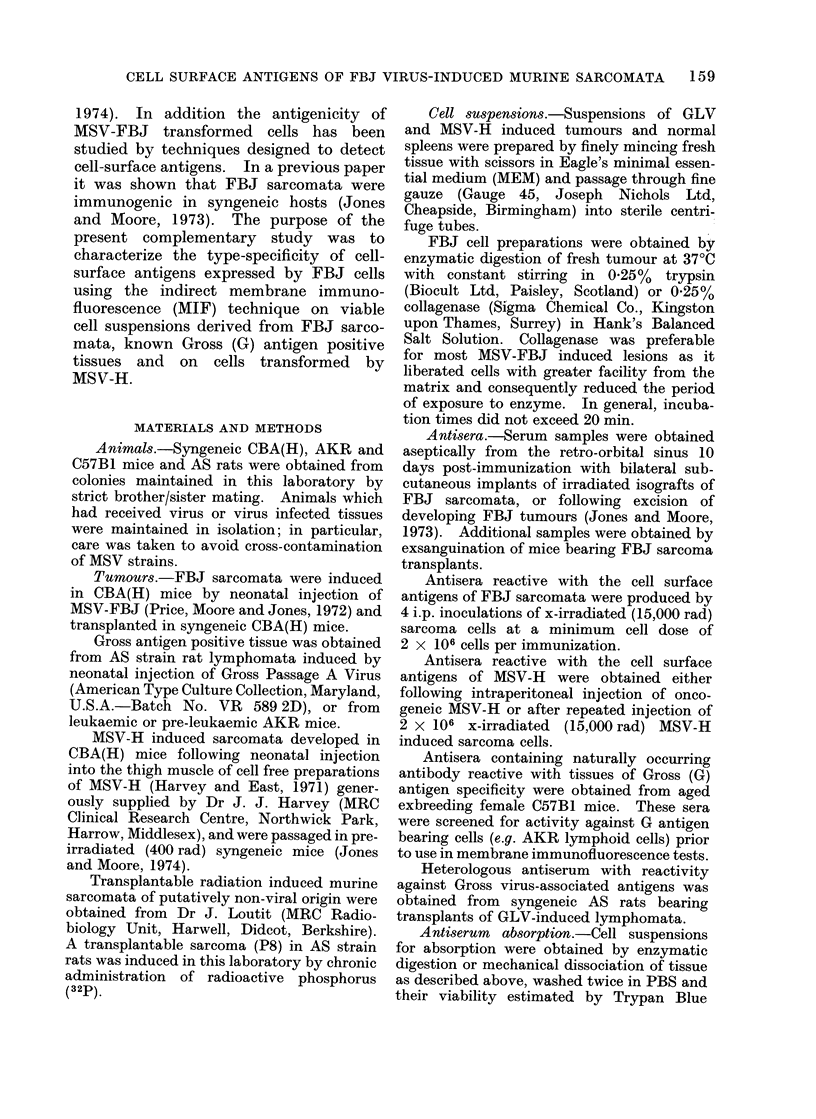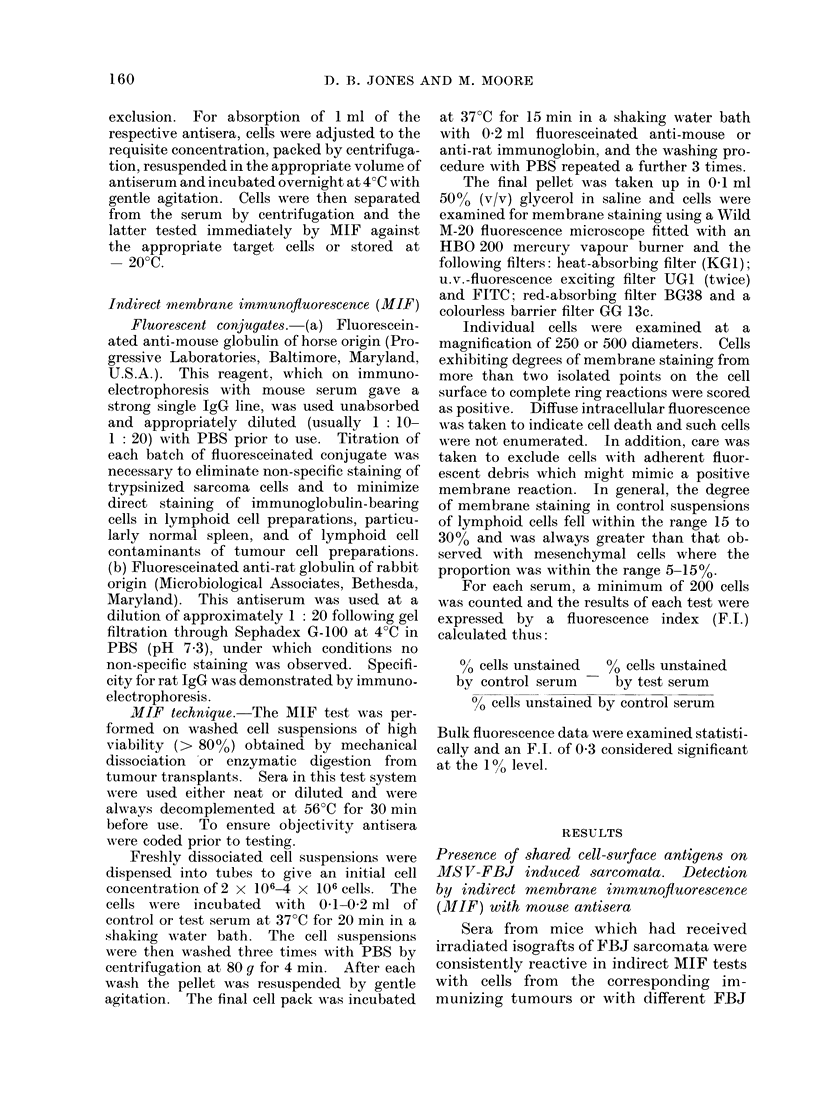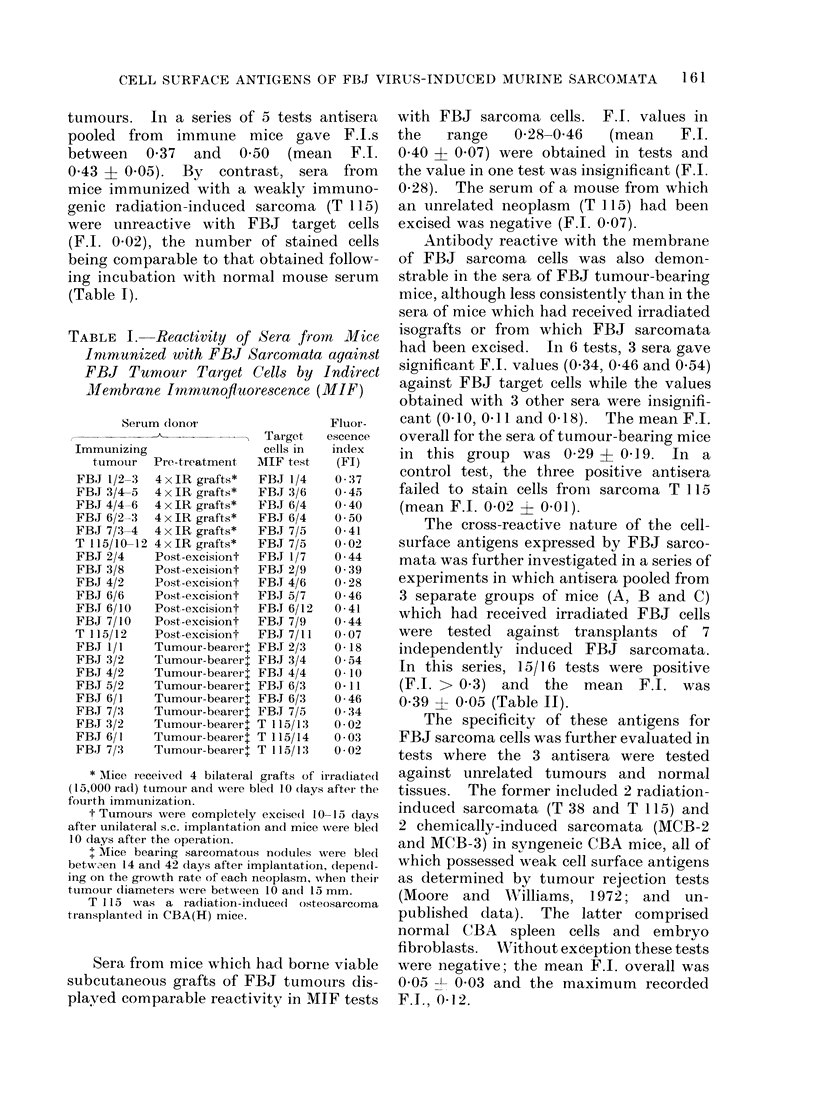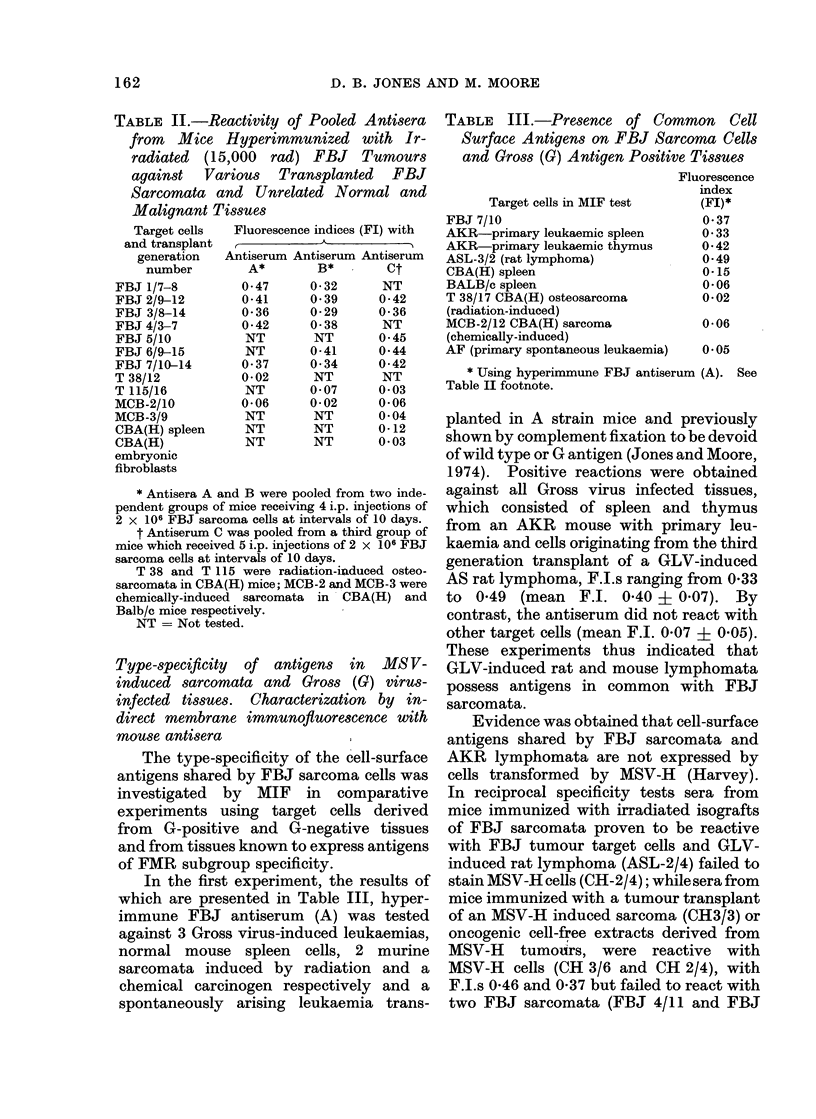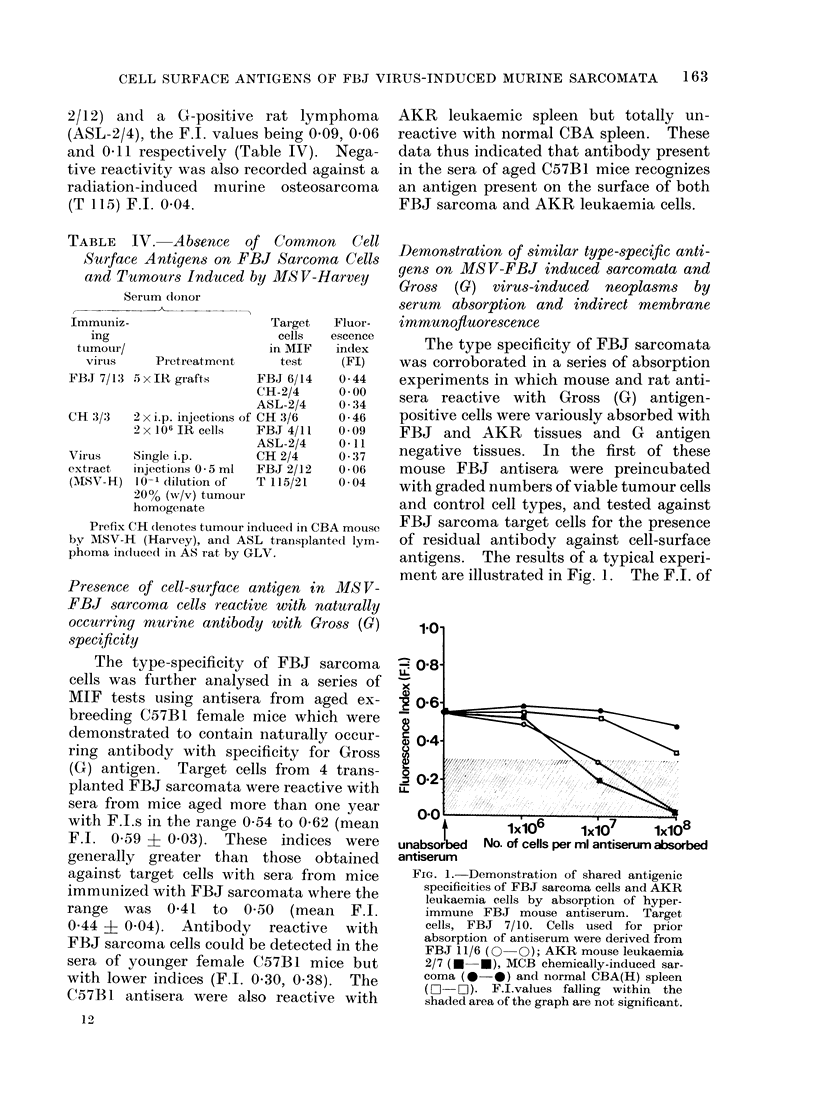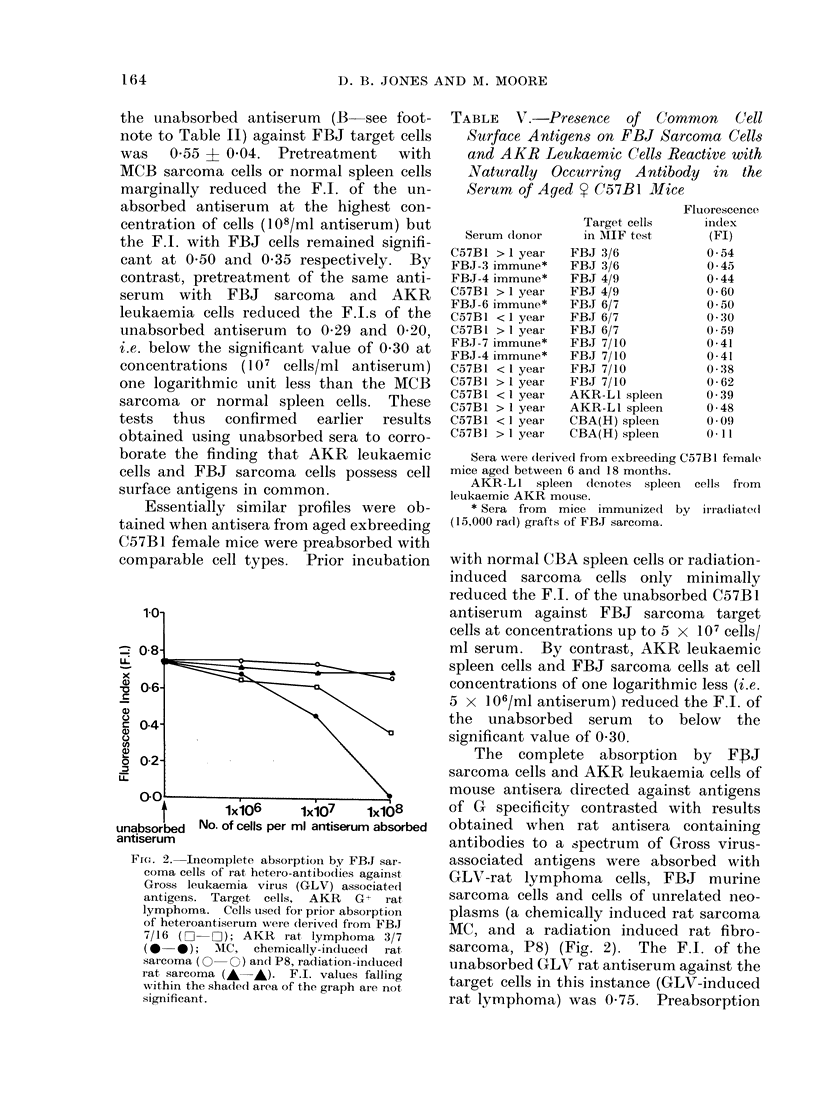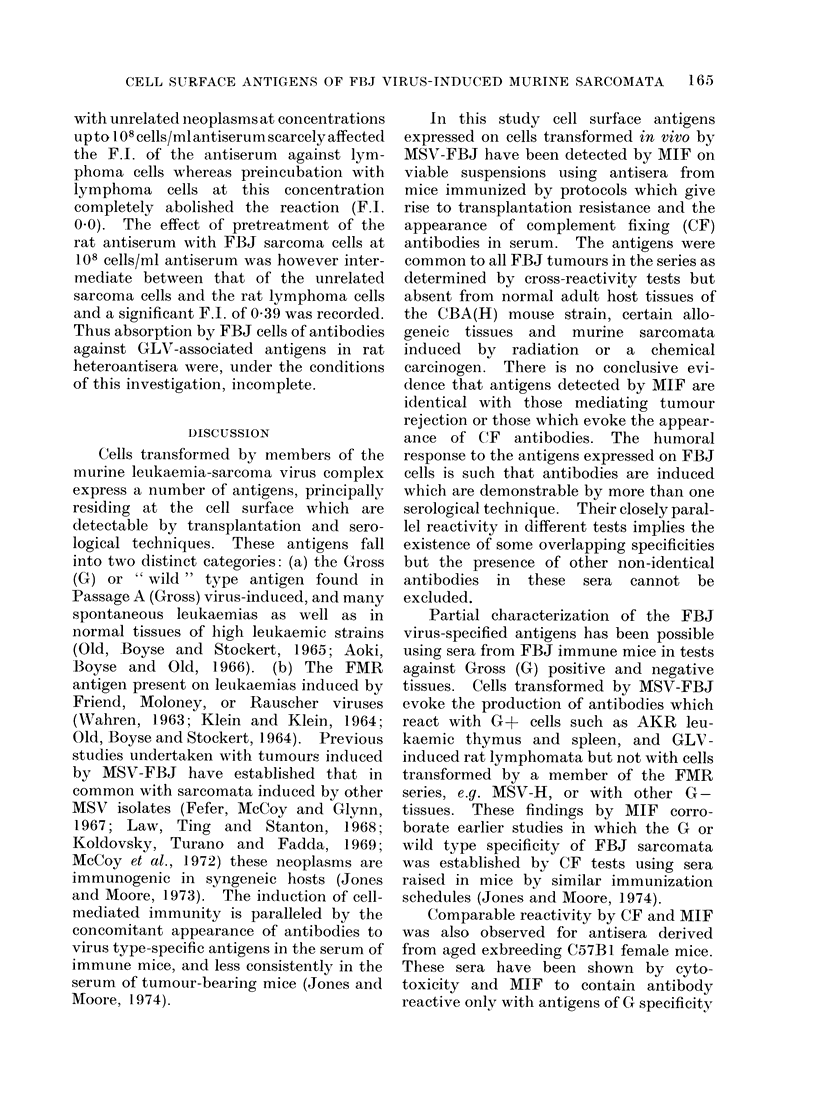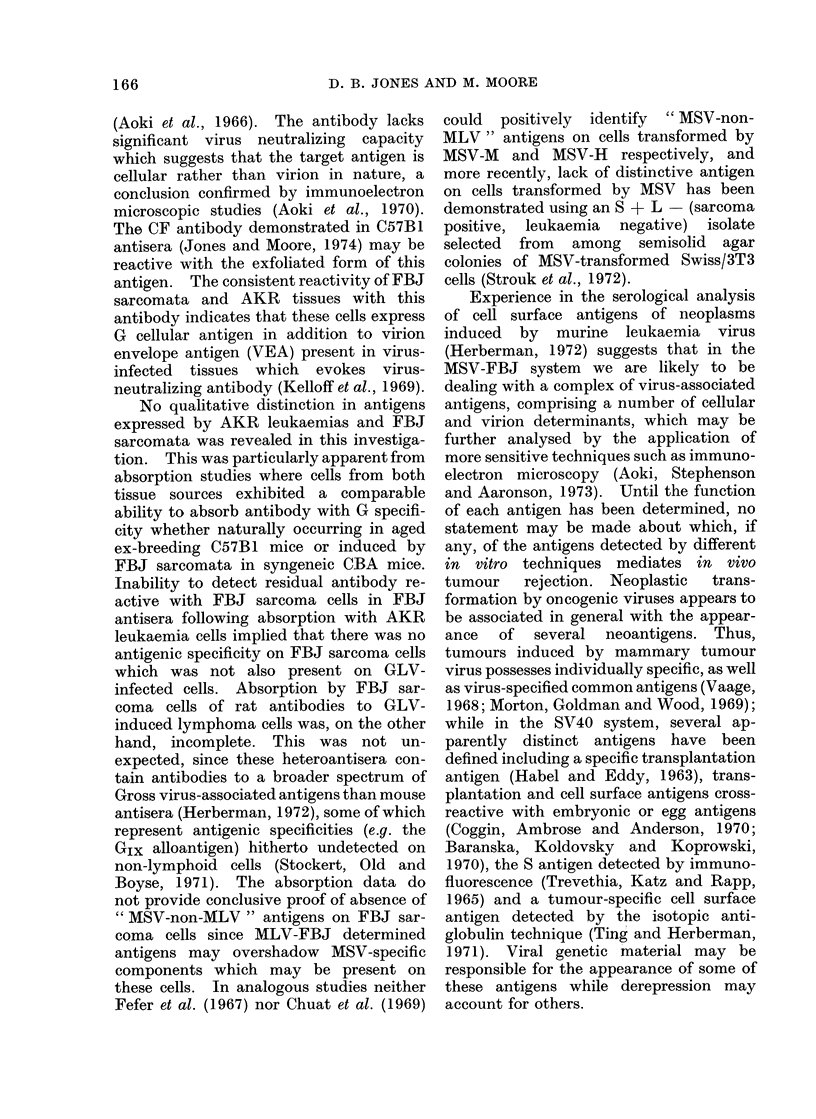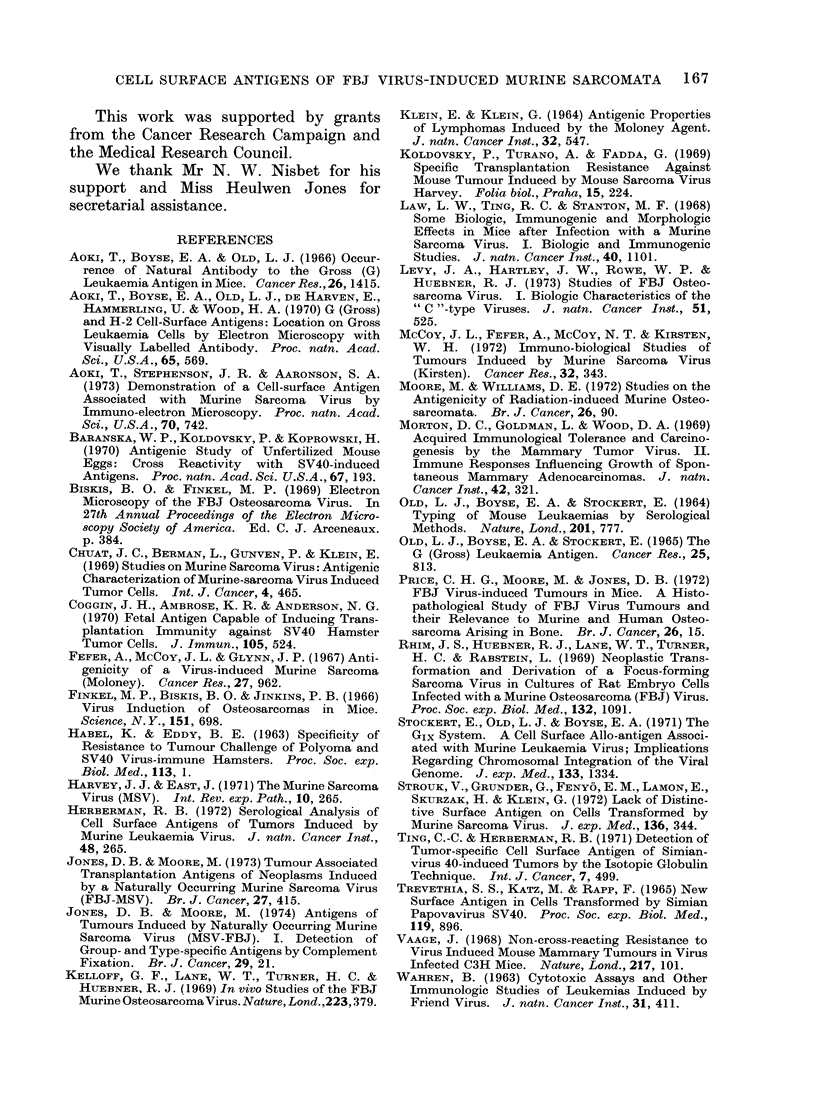# Antigens of Tumours Induced by Naturally Occurring Murine Sarcoma Virus (MSV-FBJ)

**DOI:** 10.1038/bjc.1974.52

**Published:** 1974-02

**Authors:** D. B. Jones, M. Moore

## Abstract

Cell surface antigens expressed by cells transformed *in vivo* by FBJ virus, a wild type murine sarcoma virus (MSV) complex derived from a spontaneously arising sarcoma in a CF1 mouse, have been studied by indirect membrane immunofluorescence (MIF). Using mouse antisera raised by immunization of syngeneic CBA mice with transplanted FBJ sarcomata an antigen common to all FBJ tumours was detected which was also present on Gross (G) antigen positive tissues, *viz.* leukaemic and preleukaemic AKR lymphoid cells, but absent from the tissues of mice of G negative strains. Failure to demonstrate antigenic cross-reactivity in reciprocal MIF tests using FBJ immune sera and antisera to MSV-H (Harvey), an MSV isolate of Friend-Moloney-Rauscher (FMR) sub-group specificity, established the virus type-specificity of antigens expressed by sarcoma cells transformed by the respective MSV.

The presence of a cellular antigen with G specificity on FBJ sarcoma cells was confirmed in tests with aged exbreeding C57B1 antisera containing naturally occurring G antibody lacking significant virus neutralizing activity. However, evidence for a “sarcoma-non-leukaemia” antigen on cells transformed by MSV-FBJ was not obtained since absorption studies failed to reveal any specificity on FBJ sarcoma cells which was not also present on AKR leukaemic tissues.

It is suggested that the major humoral component of the immune response to FBJ sarcoma cells is evoked against antigens specified by the associated non-pathogenic leukaemia virus (MLV-FBJ) and the relationship of antigens demonstrated by MIF to those detected previously by complement fixation (CF) and tumour rejection tests is discussed.


*Br. J. Cancer* (1974) **29**, 158

### ANTIGENS OF TUMOURS INDUCED BY NATURALLY OCCURRING MURINE SARCOMA VIRUS (MSV-FBJ)

### II. DETECTION OF CELL-SURFACE ANTIGENS BY INDIRECT MEMBRANE IMMUNOFLUORESCENCE

D. B. JONES* AND M. MOORE†

*From the Charles Salt Research Centre, Robert Jones and Agnes Hunt Orthopaedic Hospital, Oswestry, Shropshire, SY10 7AG, England*

Received 1 November 1973. Accepted 21 November 1973

**Summary.—Cell surface antigens expressed by cells transformed *in vivo* by FBJ virus, a wild type murine sarcoma virus (MSV) complex derived from a spontaneously arising sarcoma in a CF1 mouse, have been studied by indirect membrane immunofluorescence (MIF). Using mouse antisera raised by immunization of syngeneic CBA mice with transplanted FBJ sarcomata an antigen common to all FBJ tumours was detected which was also present on Gross (G) antigen positive tissues, *viz.* leukaemic and preleukaemic AKR lymphoid cells, but absent from the tissues of mice of G negative strains. Failure to demonstrate antigenic cross-reactivity in reciprocal MIF tests using FBJ immune sera and antisera to MSV-H (Harvey), an MSV isolate of Friend-Moloney-Rauscher (FMR) sub-group specificity, established the virus type-specificity of antigens expressed by sarcoma cells transformed by the respective MSV.**

**The presence of a cellular antigen with G specificity on FBJ sarcoma cells was confirmed in tests with aged exbreeding C57Bl antisera containing naturally occurring G antibody lacking significant virus neutralizing activity. However, evidence for a " sarcoma-non-leukaemia " antigen on cells transformed by MSV-FBJ was not obtained since absorption studies failed to reveal any specificity on FBJ sarcoma cells which was not also present on AKR leukaemic tissues.**

**It is suggested that the major humoral component of the immune response to FBJ sarcoma cells is evoked against antigens specified by the associated non-pathogenic leukaemia virus (MLV-FBJ) and the relationship of antigens demonstrated by MIF to those detected previously by complement fixation (CF) and tumour rejection tests is discussed.**

FBJ VIRUS, a murine sarcoma virus (MSV-FBJ) isolated in association with a non-pathogenic murine leukaemia virus (MLV-FBJ) from a spontaneously arising sarcoma in a CF1 mouse (Finkel, Biskis and Jinkins, 1966), bears a close morphological and biochemical relationship to other members of the RNA murine leukaemia-sarcoma virus complex (Biskis and Finkel, 1969; Rhim *et al.*, 1969; Levy *et al.*, 1973). Immunological studies have established that the FBJ viruses possess the group-specific (gs) antigens of murine leukaemia virus (MLV) and the type specificity of the Gross (G) or wild type subgroup of murine oncorna-viruses, by contrast with other MSV isolates (*e.g.* MSV-Harvey) of Friend-Moloney-Rauscher (FMR) subgroup specificity (Kelloff *et al.*, 1969; Jones and Moore,

* Present address (for reprints): Department of Experimental Pathology and Morbid Anatomy, University of Southampton, Faculty of Medicine, Southampton General Hospital, Tremona Road, Southampton SO9 4XY.

† Present address: Immunology Department, Paterson Laboratories, Christie Hospital and Holt Radium Institute, Manchester M20 9BX.

1974). In addition the antigenicity of MSV-FBJ transformed cells has been studied by techniques designed to detect cell-surface antigens. In a previous paper it was shown that FBJ sarcomata were immunogenic in syngeneic hosts (Jones and Moore, 1973). The purpose of the present complementary study was to characterize the type-specificity of cell-surface antigens expressed by FBJ cells using the indirect membrane immunofluorescence (MIF) technique on viable cell suspensions derived from FBJ sarcomata, known Gross (G) antigen positive tissues and on cells transformed by MSV-H.

### MATERIALS AND METHODS

*Animals.*—Syngeneic CBA(H), AKR and C57B1 mice and AS rats were obtained from colonies maintained in this laboratory by strict brother/sister mating. Animals which had received virus or virus infected tissues were maintained in isolation; in particular, care was taken to avoid cross-contamination of MSV strains.

*Tumours.*—FBJ sarcomata were induced in CBA(H) mice by neonatal injection of MSV-FBJ (Price, Moore and Jones, 1972) and transplanted in syngeneic CBA(H) mice.

Gross antigen positive tissue was obtained from AS strain rat lymphomata induced by neonatal injection of Gross Passage A Virus (American Type Culture Collection, Maryland, U.S.A.—Batch No. VR 589 2D), or from leukaemic or pre-leukaemic AKR mice.

MSV-H induced sarcomata developed in CBA(H) mice following neonatal injection into the thigh muscle of cell free preparations of MSV-H (Harvey and East, 1971) generously supplied by Dr J. J. Harvey (MRC Clinical Research Centre, Northwick Park, Harrow, Middlesex), and were passaged in pre-irradiated (400 rad) syngeneic mice (Jones and Moore, 1974).

Transplantable radiation induced murine sarcomata of putatively non-viral origin were obtained from Dr J. Loutit (MRC Radiobiology Unit, Harwell, Didcot, Berkshire). A transplantable sarcoma (P8) in AS strain rats was induced in this laboratory by chronic administration of radioactive phosphorus ($^{32}P$).

*Cell suspensions.*—Suspensions of GLV and MSV-H induced tumours and normal spleens were prepared by finely mincing fresh tissue with scissors in Eagle's minimal essential medium (MEM) and passage through fine gauze (Gauge 45, Joseph Nichols Ltd, Cheapside, Birmingham) into sterile centrifuge tubes.

FBJ cell preparations were obtained by enzymatic digestion of fresh tumour at 37°C with constant stirring in 0·25% trypsin (Biocult Ltd, Paisley, Scotland) or 0·25% collagenase (Sigma Chemical Co., Kingston upon Thames, Surrey) in Hank's Balanced Salt Solution. Collagenase was preferable for most MSV-FBJ induced lesions as it liberated cells with greater facility from the matrix and consequently reduced the period of exposure to enzyme. In general, incubation times did not exceed 20 min.

*Antisera.*—Serum samples were obtained aseptically from the retro-orbital sinus 10 days post-immunization with bilateral subcutaneous implants of irradiated isografts of FBJ sarcomata, or following excision of developing FBJ tumours (Jones and Moore, 1973). Additional samples were obtained by exsanguination of mice bearing FBJ sarcoma transplants.

Antisera reactive with the cell surface antigens of FBJ sarcomata were produced by 4 i.p. inoculations of x-irradiated (15,000 rad) sarcoma cells at a minimum cell dose of $2 \times 10^6$ cells per immunization.

Antisera reactive with the cell surface antigens of MSV-H were obtained either following intraperitoneal injection of oncogeneic MSV-H or after repeated injection of $2 \times 10^6$ x-irradiated (15,000 rad) MSV-H induced sarcoma cells.

Antisera containing naturally occurring antibody reactive with tissues of Gross (G) antigen specificity were obtained from aged exbreeding female C57B1 mice. These sera were screened for activity against G antigen bearing cells (*e.g.* AKR lymphoid cells) prior to use in membrane immunofluorescence tests.

Heterologous antiserum with reactivity against Gross virus-associated antigens was obtained from syngeneic AS rats bearing transplants of GLV-induced lymphomata.

*Antiserum absorption.*—Cell suspensions for absorption were obtained by enzymatic digestion or mechanical dissociation of tissue as described above, washed twice in PBS and their viability estimated by Trypan Blue exclusion. For absorption of 1 ml of the respective antisera, cells were adjusted to the requisite concentration, packed by centrifugation, resuspended in the appropriate volume of antiserum and incubated overnight at 4°C with gentle agitation. Cells were then separated from the serum by centrifugation and the latter tested immediately by MIF against the appropriate target cells or stored at − 20°C.

*Indirect membrane immunofluorescence* (*MIF*)

*Fluorescent conjugates.*—(a) Fluoresceinated anti-mouse globulin of horse origin (Progressive Laboratories, Baltimore, Maryland, U.S.A.). This reagent, which on immunoelectrophoresis with mouse serum gave a strong single IgG line, was used unabsorbed and appropriately diluted (usually 1 : 10–1 : 20) with PBS prior to use. Titration of each batch of fluoresceinated conjugate was necessary to eliminate non-specific staining of trypsinized sarcoma cells and to minimize direct staining of immunoglobulin-bearing cells in lymphoid cell preparations, particularly normal spleen, and of lymphoid cell contaminants of tumour cell preparations. (b) Fluoresceinated anti-rat globulin of rabbit origin (Microbiological Associates, Bethesda, Maryland). This antiserum was used at a dilution of approximately 1 : 20 following gel filtration through Sephadex G-100 at 4°C in PBS (pH 7·3), under which conditions no non-specific staining was observed. Specificity for rat IgG was demonstrated by immunoelectrophoresis.

*MIF technique.*—The MIF test was performed on washed cell suspensions of high viability (> 80%) obtained by mechanical dissociation or enzymatic digestion from tumour transplants. Sera in this test system were used either neat or diluted and were always decomplemented at 56°C for 30 min before use. To ensure objectivity antisera were coded prior to testing.

Freshly dissociated cell suspensions were dispensed into tubes to give an initial cell concentration of $2 \times 10^6$–$4 \times 10^6$ cells. The cells were incubated with 0·1–0·2 ml of control or test serum at 37°C for 20 min in a shaking water bath. The cell suspensions were then washed three times with PBS by centrifugation at 80 *g* for 4 min. After each wash the pellet was resuspended by gentle agitation. The final cell pack was incubated at 37°C for 15 min in a shaking water bath with 0·2 ml fluoresceinated anti-mouse or anti-rat immunoglobin, and the washing procedure with PBS repeated a further 3 times.

The final pellet was taken up in 0·1 ml 50% (v/v) glycerol in saline and cells were examined for membrane staining using a Wild M-20 fluorescence microscope fitted with an HBO 200 mercury vapour burner and the following filters: heat-absorbing filter (KG1); u.v.-fluorescence exciting filter UG1 (twice) and FITC; red-absorbing filter BG38 and a colourless barrier filter GG 13c.

Individual cells were examined at a magnification of 250 or 500 diameters. Cells exhibiting degrees of membrane staining from more than two isolated points on the cell surface to complete ring reactions were scored as positive. Diffuse intracellular fluorescence was taken to indicate cell death and such cells were not enumerated. In addition, care was taken to exclude cells with adherent fluorescent debris which might mimic a positive membrane reaction. In general, the degree of membrane staining in control suspensions of lymphoid cells fell within the range 15 to 30% and was always greater than that observed with mesenchymal cells where the proportion was within the range 5–15%.

For each serum, a minimum of 200 cells was counted and the results of each test were expressed by a fluorescence index (F.I.) calculated thus:

$$\frac{\begin{array}{c}\%\text{ cells unstained}\\ \text{by control serum}\end{array} - \begin{array}{c}\%\text{ cells unstained}\\ \text{by test serum}\end{array}}{\%\text{ cells unstained by control serum}}$$

Bulk fluorescence data were examined statistically and an F.I. of 0·3 considered significant at the 1% level.

### RESULTS

*Presence of shared cell-surface antigens on MSV-FBJ induced sarcomata. Detection by indirect membrane immunofluorescence (MIF) with mouse antisera*

Sera from mice which had received irradiated isografts of FBJ sarcomata were consistently reactive in indirect MIF tests with cells from the corresponding immunizing tumours or with different FBJ

tumours. In a series of 5 tests antisera pooled from immune mice gave F.I.s between 0·37 and 0·50 (mean F.I. 0·43 ± 0·05). By contrast, sera from mice immunized with a weakly immunogenic radiation-induced sarcoma (T 115) were unreactive with FBJ target cells (F.I. 0·02), the number of stained cells being comparable to that obtained following incubation with normal mouse serum (Table I).

TABLE I.—*Reactivity of Sera from Mice Immunized with FBJ Sarcomata against FBJ Tumour Target Cells by Indirect Membrane Immunofluorescence (MIF)*

| Serum donor: Immunizing tumour | Serum donor: Pre-treatment | Target cells in MIF test | Fluorescence index (FI) |
|---|---|---|---|
| FBJ 1/2–3 | 4×IR grafts* | FBJ 1/4 | 0·37 |
| FBJ 3/4–5 | 4×IR grafts* | FBJ 3/6 | 0·45 |
| FBJ 4/4–6 | 4×IR grafts* | FBJ 6/4 | 0·40 |
| FBJ 6/2–3 | 4×IR grafts* | FBJ 6/4 | 0·50 |
| FBJ 7/3–4 | 4×IR grafts* | FBJ 7/5 | 0·41 |
| T 115/10–12 | 4×IR grafts* | FBJ 7/5 | 0·02 |
| FBJ 2/4 | Post-excision† | FBJ 1/7 | 0·44 |
| FBJ 3/8 | Post-excision† | FBJ 2/9 | 0·39 |
| FBJ 4/2 | Post-excision† | FBJ 4/6 | 0·28 |
| FBJ 6/6 | Post-excision† | FBJ 5/7 | 0·46 |
| FBJ 6/10 | Post-excision† | FBJ 6/12 | 0·41 |
| FBJ 7/10 | Post-excision† | FBJ 7/9 | 0·44 |
| T 115/12 | Post-excision† | FBJ 7/11 | 0·07 |
| FBJ 1/1 | Tumour-bearer‡ | FBJ 2/3 | 0·18 |
| FBJ 3/2 | Tumour-bearer‡ | FBJ 3/4 | 0·54 |
| FBJ 4/2 | Tumour-bearer‡ | FBJ 4/4 | 0·10 |
| FBJ 5/2 | Tumour-bearer‡ | FBJ 6/3 | 0·11 |
| FBJ 6/1 | Tumour-bearer‡ | FBJ 6/3 | 0·46 |
| FBJ 7/3 | Tumour-bearer‡ | FBJ 7/5 | 0·34 |
| FBJ 3/2 | Tumour-bearer‡ | T 115/13 | 0·02 |
| FBJ 6/1 | Tumour-bearer‡ | T 115/14 | 0·03 |
| FBJ 7/3 | Tumour-bearer‡ | T 115/13 | 0·02 |

* Mice received 4 bilateral grafts of irradiated (15,000 rad) tumour and were bled 10 days after the fourth immunization.

† Tumours were completely excised 10–15 days after unilateral s.c. implantation and mice were bled 10 days after the operation.

‡ Mice bearing sarcomatous nodules were bled between 14 and 42 days after implantation, depending on the growth rate of each neoplasm, when their tumour diameters were between 10 and 15 mm.

T 115 was a radiation-induced osteosarcoma transplanted in CBA(H) mice.

Sera from mice which had borne viable subcutaneous grafts of FBJ tumours displayed comparable reactivity in MIF tests with FBJ sarcoma cells. F.I. values in the range 0·28–0·46 (mean F.I. 0·40 ± 0·07) were obtained in tests and the value in one test was insignificant (F.I. 0·28). The serum of a mouse from which an unrelated neoplasm (T 115) had been excised was negative (F.I. 0·07).

Antibody reactive with the membrane of FBJ sarcoma cells was also demonstrable in the sera of FBJ tumour-bearing mice, although less consistently than in the sera of mice which had received irradiated isografts or from which FBJ sarcomata had been excised. In 6 tests, 3 sera gave significant F.I. values (0·34, 0·46 and 0·54) against FBJ target cells while the values obtained with 3 other sera were insignificant (0·10, 0·11 and 0·18). The mean F.I. overall for the sera of tumour-bearing mice in this group was 0·29 ± 0·19. In a control test, the three positive antisera failed to stain cells from sarcoma T 115 (mean F.I. 0·02 ± 0·01).

The cross-reactive nature of the cell-surface antigens expressed by FBJ sarcomata was further investigated in a series of experiments in which antisera pooled from 3 separate groups of mice (A, B and C) which had received irradiated FBJ cells were tested against transplants of 7 independently induced FBJ sarcomata. In this series, 15/16 tests were positive (F.I. > 0·3) and the mean F.I. was 0·39 ± 0·05 (Table II).

The specificity of these antigens for FBJ sarcoma cells was further evaluated in tests where the 3 antisera were tested against unrelated tumours and normal tissues. The former included 2 radiation-induced sarcomata (T 38 and T 115) and 2 chemically-induced sarcomata (MCB-2 and MCB-3) in syngeneic CBA mice, all of which possessed weak cell surface antigens as determined by tumour rejection tests (Moore and Williams, 1972; and unpublished data). The latter comprised normal CBA spleen cells and embryo fibroblasts. Without exception these tests were negative; the mean F.I. overall was 0·05 ± 0·03 and the maximum recorded F.I., 0·12.

TABLE II.—*Reactivity of Pooled Antisera from Mice Hyperimmunized with Irradiated (15,000 rad) FBJ Tumours against Various Transplanted FBJ Sarcomata and Unrelated Normal and Malignant Tissues*

| Target cells and transplant generation number | Fluorescence indices (FI) with | | |
|---|---|---|---|
| | Antiserum A* | Antiserum B* | Antiserum C† |
| FBJ 1/7–8 | 0·47 | 0·32 | NT |
| FBJ 2/9–12 | 0·41 | 0·39 | 0·42 |
| FBJ 3/8–14 | 0·36 | 0·29 | 0·36 |
| FBJ 4/3–7 | 0·42 | 0·38 | NT |
| FBJ 5/10 | NT | NT | 0·45 |
| FBJ 6/9–15 | NT | 0·41 | 0·44 |
| FBJ 7/10–14 | 0·37 | 0·34 | 0·42 |
| T 38/12 | 0·02 | NT | NT |
| T 115/16 | NT | 0·07 | 0·03 |
| MCB-2/10 | 0·06 | 0·02 | 0·06 |
| MCB-3/9 | NT | NT | 0·04 |
| CBA(H) spleen | NT | NT | 0·12 |
| CBA(H) embryonic fibroblasts | NT | NT | 0·03 |

* Antisera A and B were pooled from two independent groups of mice receiving 4 i.p. injections of $2 \times 10^6$ FBJ sarcoma cells at intervals of 10 days.

† Antiserum C was pooled from a third group of mice which received 5 i.p. injections of $2 \times 10^6$ FBJ sarcoma cells at intervals of 10 days.

T 38 and T 115 were radiation-induced osteosarcomata in CBA(H) mice; MCB-2 and MCB-3 were chemically-induced sarcomata in CBA(H) and Balb/c mice respectively.

NT = Not tested.

*Type-specificity of antigens in MSV-induced sarcomata and Gross (G) virus-infected tissues. Characterization by indirect membrane immunofluorescence with mouse antisera*

The type-specificity of the cell-surface antigens shared by FBJ sarcoma cells was investigated by MIF in comparative experiments using target cells derived from G-positive and G-negative tissues and from tissues known to express antigens of FMR subgroup specificity.

In the first experiment, the results of which are presented in Table III, hyperimmune FBJ antiserum (A) was tested against 3 Gross virus-induced leukaemias, normal mouse spleen cells, 2 murine sarcomata induced by radiation and a chemical carcinogen respectively and a spontaneously arising leukaemia transplanted in A strain mice and previously shown by complement fixation to be devoid of wild type or G antigen (Jones and Moore, 1974). Positive reactions were obtained against all Gross virus infected tissues, which consisted of spleen and thymus from an AKR mouse with primary leukaemia and cells originating from the third generation transplant of a GLV-induced AS rat lymphoma, F.I.s ranging from 0·33 to 0·49 (mean F.I. 0·40 ± 0·07). By contrast, the antiserum did not react with other target cells (mean F.I. 0·07 ± 0·05). These experiments thus indicated that GLV-induced rat and mouse lymphomata possess antigens in common with FBJ sarcomata.

TABLE III.—*Presence of Common Cell Surface Antigens on FBJ Sarcoma Cells and Gross (G) Antigen Positive Tissues*

| Target cells in MIF test | Fluorescence index (FI)* |
|---|---|
| FBJ 7/10 | 0·37 |
| AKR—primary leukaemic spleen | 0·33 |
| AKR—primary leukaemic thymus | 0·42 |
| ASL-3/2 (rat lymphoma) | 0·49 |
| CBA(H) spleen | 0·15 |
| BALB/c spleen | 0·06 |
| T 38/17 CBA(H) osteosarcoma (radiation-induced) | 0·02 |
| MCB-2/12 CBA(H) sarcoma (chemically-induced) | 0·06 |
| AF (primary spontaneous leukaemia) | 0·05 |

* Using hyperimmune FBJ antiserum (A). See Table II footnote.

Evidence was obtained that cell-surface antigens shared by FBJ sarcomata and AKR lymphomata are not expressed by cells transformed by MSV-H (Harvey). In reciprocal specificity tests sera from mice immunized with irradiated isografts of FBJ sarcomata proven to be reactive with FBJ tumour target cells and GLV-induced rat lymphoma (ASL-2/4) failed to stain MSV-H cells (CH-2/4); while sera from mice immunized with a tumour transplant of an MSV-H induced sarcoma (CH3/3) or oncogenic cell-free extracts derived from MSV-H tumours, were reactive with MSV-H cells (CH 3/6 and CH 2/4), with F.I.s 0·46 and 0·37 but failed to react with two FBJ sarcomata (FBJ 4/11 and FBJ

2/12) and a G-positive rat lymphoma (ASL-2/4), the F.I. values being 0·09, 0·06 and 0·11 respectively (Table IV). Negative reactivity was also recorded against a radiation-induced murine osteosarcoma (T 115) F.I. 0·04.

TABLE IV.—*Absence of Common Cell Surface Antigens on FBJ Sarcoma Cells and Tumours Induced by MSV-Harvey*

| Serum donor: Immunizing tumour/virus | Serum donor: Pretreatment | Target cells in MIF test | Fluorescence index (FI) |
|---|---|---|---|
| FBJ 7/13 | 5×IR grafts | FBJ 6/14 | 0·44 |
| | | CH-2/4 | 0·00 |
| | | ASL-2/4 | 0·34 |
| CH 3/3 | 2×i.p. injections of $2\times10^6$ IR cells | CH 3/6 | 0·46 |
| | | FBJ 4/11 | 0·09 |
| | | ASL-2/4 | 0·11 |
| Virus extract (MSV-H) | Single i.p. injections 0·5 ml $10^{-1}$ dilution of 20% (w/v) tumour homogenate | CH 2/4 | 0·37 |
| | | FBJ 2/12 | 0·06 |
| | | T 115/21 | 0·04 |

Prefix CH denotes tumour induced in CBA mouse by MSV-H (Harvey), and ASL transplanted lymphoma induced in AS rat by GLV.

*Presence of cell-surface antigen in MSV-FBJ sarcoma cells reactive with naturally occurring murine antibody with Gross (G) specificity*

The type-specificity of FBJ sarcoma cells was further analysed in a series of MIF tests using antisera from aged ex-breeding C57Bl female mice which were demonstrated to contain naturally occurring antibody with specificity for Gross (G) antigen. Target cells from 4 transplanted FBJ sarcomata were reactive with sera from mice aged more than one year with F.I.s in the range 0·54 to 0·62 (mean F.I. 0·59 ± 0·03). These indices were generally greater than those obtained against target cells with sera from mice immunized with FBJ sarcomata where the range was 0·41 to 0·50 (mean F.I. 0·44 ± 0·04). Antibody reactive with FBJ sarcoma cells could be detected in the sera of younger female C57Bl mice but with lower indices (F.I. 0·30, 0·38). The C57Bl antisera were also reactive with AKR leukaemic spleen but totally unreactive with normal CBA spleen. These data thus indicated that antibody present in the sera of aged C57Bl mice recognizes an antigen present on the surface of both FBJ sarcoma and AKR leukaemia cells.

*Demonstration of similar type-specific antigens on MSV-FBJ induced sarcomata and Gross (G) virus-induced neoplasms by serum absorption and indirect membrane immunofluorescence*

The type specificity of FBJ sarcomata was corroborated in a series of absorption experiments in which mouse and rat antisera reactive with Gross (G) antigen-positive cells were variously absorbed with FBJ and AKR tissues and G antigen negative tissues. In the first of these mouse FBJ antisera were preincubated with graded numbers of viable tumour cells and control cell types, and tested against FBJ sarcoma target cells for the presence of residual antibody against cell-surface antigens. The results of a typical experiment are illustrated in Fig. 1. The F.I. of

FIG. 1.—Demonstration of shared antigenic specificities of FBJ sarcoma cells and AKR leukaemia cells by absorption of hyperimmune FBJ mouse antiserum. Target cells, FBJ 7/10. Cells used for prior absorption of antiserum were derived from FBJ 11/6 (○—○); AKR mouse leukaemia 2/7 (■—■), MCB chemically-induced sarcoma (●—●) and normal CBA(H) spleen (□—□). F.I.values falling within the shaded area of the graph are not significant.

the unabsorbed antiserum (B—see footnote to Table II) against FBJ target cells was 0·55 ± 0·04. Pretreatment with MCB sarcoma cells or normal spleen cells marginally reduced the F.I. of the unabsorbed antiserum at the highest concentration of cells ($10^8$/ml antiserum) but the F.I. with FBJ cells remained significant at 0·50 and 0·35 respectively. By contrast, pretreatment of the same antiserum with FBJ sarcoma and AKR leukaemia cells reduced the F.I.s of the unabsorbed antiserum to 0·29 and 0·20, *i.e.* below the significant value of 0·30 at concentrations ($10^7$ cells/ml antiserum) one logarithmic unit less than the MCB sarcoma or normal spleen cells. These tests thus confirmed earlier results obtained using unabsorbed sera to corroborate the finding that AKR leukaemic cells and FBJ sarcoma cells possess cell surface antigens in common.

Essentially similar profiles were obtained when antisera from aged exbreeding C57B1 female mice were preabsorbed with comparable cell types. Prior incubation

FIG. 2.—Incomplete absorption by FBJ sarcoma cells of rat hetero-antibodies against Gross leukaemia virus (GLV) associated antigens. Target cells, AKR $G^+$ rat lymphoma. Cells used for prior absorption of heteroantiserum were derived from FBJ 7/16 (□—□); AKR rat lymphoma 3/7 (●—●); MC, chemically-induced rat sarcoma (○—○) and P8, radiation-induced rat sarcoma (▲—▲). F.I. values falling within the shaded area of the graph are not significant.

TABLE V.—*Presence of Common Cell Surface Antigens on FBJ Sarcoma Cells and AKR Leukaemic Cells Reactive with Naturally Occurring Antibody in the Serum of Aged ♀ C57B1 Mice*

| Serum donor | Target cells in MIF test | Fluorescence index (FI) |
|---|---|---|
| C57B1 > 1 year | FBJ 3/6 | 0·54 |
| FBJ-3 immune* | FBJ 3/6 | 0·45 |
| FBJ-4 immune* | FBJ 4/9 | 0·44 |
| C57B1 > 1 year | FBJ 4/9 | 0·60 |
| FBJ-6 immune* | FBJ 6/7 | 0·50 |
| C57B1 < 1 year | FBJ 6/7 | 0·30 |
| C57B1 > 1 year | FBJ 6/7 | 0·59 |
| FBJ-7 immune* | FBJ 7/10 | 0·41 |
| FBJ-4 immune* | FBJ 7/10 | 0·41 |
| C57B1 < 1 year | FBJ 7/10 | 0·38 |
| C57B1 > 1 year | FBJ 7/10 | 0·62 |
| C57B1 < 1 year | AKR-L1 spleen | 0·39 |
| C57B1 > 1 year | AKR-L1 spleen | 0·48 |
| C57B1 < 1 year | CBA(H) spleen | 0·09 |
| C57B1 > 1 year | CBA(H) spleen | 0·11 |

Sera were derived from exbreeding C57B1 female mice aged between 6 and 18 months.

AKR-L1 spleen denotes spleen cells from leukaemic AKR mouse.

* Sera from mice immunized by irradiated (15,000 rad) grafts of FBJ sarcoma.

with normal CBA spleen cells or radiation-induced sarcoma cells only minimally reduced the F.I. of the unabsorbed C57B1 antiserum against FBJ sarcoma target cells at concentrations up to 5 × $10^7$ cells/ml serum. By contrast, AKR leukaemic spleen cells and FBJ sarcoma cells at cell concentrations of one logarithmic less (*i.e.* 5 × $10^6$/ml antiserum) reduced the F.I. of the unabsorbed serum to below the significant value of 0·30.

The complete absorption by FBJ sarcoma cells and AKR leukaemia cells of mouse antisera directed against antigens of G specificity contrasted with results obtained when rat antisera containing antibodies to a spectrum of Gross virus-associated antigens were absorbed with GLV-rat lymphoma cells, FBJ murine sarcoma cells and cells of unrelated neoplasms (a chemically induced rat sarcoma MC, and a radiation induced rat fibrosarcoma, P8) (Fig. 2). The F.I. of the unabsorbed GLV rat antiserum against the target cells in this instance (GLV-induced rat lymphoma) was 0·75. Preabsorption with unrelated neoplasms at concentrations up to $10^8$ cells/ml antiserum scarcely affected the F.I. of the antiserum against lymphoma cells whereas preincubation with lymphoma cells at this concentration completely abolished the reaction (F.I. 0·0). The effect of pretreatment of the rat antiserum with FBJ sarcoma cells at $10^8$ cells/ml antiserum was however intermediate between that of the unrelated sarcoma cells and the rat lymphoma cells and a significant F.I. of 0·39 was recorded. Thus absorption by FBJ cells of antibodies against GLV-associated antigens in rat heteroantisera were, under the conditions of this investigation, incomplete.

### DISCUSSION

Cells transformed by members of the murine leukaemia-sarcoma virus complex express a number of antigens, principally residing at the cell surface which are detectable by transplantation and serological techniques. These antigens fall into two distinct categories: (a) the Gross (G) or "wild" type antigen found in Passage A (Gross) virus-induced, and many spontaneous leukaemias as well as in normal tissues of high leukaemic strains (Old, Boyse and Stockert, 1965; Aoki, Boyse and Old, 1966). (b) The FMR antigen present on leukaemias induced by Friend, Moloney, or Rauscher viruses (Wahren, 1963; Klein and Klein, 1964; Old, Boyse and Stockert, 1964). Previous studies undertaken with tumours induced by MSV-FBJ have established that in common with sarcomata induced by other MSV isolates (Fefer, McCoy and Glynn, 1967; Law, Ting and Stanton, 1968; Koldovsky, Turano and Fadda, 1969; McCoy *et al.*, 1972) these neoplasms are immunogenic in syngeneic hosts (Jones and Moore, 1973). The induction of cell-mediated immunity is paralleled by the concomitant appearance of antibodies to virus type-specific antigens in the serum of immune mice, and less consistently in the serum of tumour-bearing mice (Jones and Moore, 1974).

In this study cell surface antigens expressed on cells transformed *in vivo* by MSV-FBJ have been detected by MIF on viable suspensions using antisera from mice immunized by protocols which give rise to transplantation resistance and the appearance of complement fixing (CF) antibodies in serum. The antigens were common to all FBJ tumours in the series as determined by cross-reactivity tests but absent from normal adult host tissues of the CBA(H) mouse strain, certain allogeneic tissues and murine sarcomata induced by radiation or a chemical carcinogen. There is no conclusive evidence that antigens detected by MIF are identical with those mediating tumour rejection or those which evoke the appearance of CF antibodies. The humoral response to the antigens expressed on FBJ cells is such that antibodies are induced which are demonstrable by more than one serological technique. Their closely parallel reactivity in different tests implies the existence of some overlapping specificities but the presence of other non-identical antibodies in these sera cannot be excluded.

Partial characterization of the FBJ virus-specified antigens has been possible using sera from FBJ immune mice in tests against Gross (G) positive and negative tissues. Cells transformed by MSV-FBJ evoke the production of antibodies which react with G+ cells such as AKR leukaemic thymus and spleen, and GLV-induced rat lymphomata but not with cells transformed by a member of the FMR series, *e.g.* MSV-H, or with other G− tissues. These findings by MIF corroborate earlier studies in which the G or wild type specificity of FBJ sarcomata was established by CF tests using sera raised in mice by similar immunization schedules (Jones and Moore, 1974).

Comparable reactivity by CF and MIF was also observed for antisera derived from aged exbreeding C57Bl female mice. These sera have been shown by cytotoxicity and MIF to contain antibody reactive only with antigens of G specificity

(Aoki *et al.*, 1966). The antibody lacks significant virus neutralizing capacity which suggests that the target antigen is cellular rather than virion in nature, a conclusion confirmed by immunoelectron microscopic studies (Aoki *et al.*, 1970). The CF antibody demonstrated in C57B1 antisera (Jones and Moore, 1974) may be reactive with the exfoliated form of this antigen. The consistent reactivity of FBJ sarcomata and AKR tissues with this antibody indicates that these cells express G cellular antigen in addition to virion envelope antigen (VEA) present in virus-infected tissues which evokes virus-neutralizing antibody (Kelloff *et al.*, 1969).

No qualitative distinction in antigens expressed by AKR leukaemias and FBJ sarcomata was revealed in this investigation. This was particularly apparent from absorption studies where cells from both tissue sources exhibited a comparable ability to absorb antibody with G specificity whether naturally occurring in aged ex-breeding C57B1 mice or induced by FBJ sarcomata in syngeneic CBA mice. Inability to detect residual antibody reactive with FBJ sarcoma cells in FBJ antisera following absorption with AKR leukaemia cells implied that there was no antigenic specificity on FBJ sarcoma cells which was not also present on GLV-infected cells. Absorption by FBJ sarcoma cells of rat antibodies to GLV-induced lymphoma cells was, on the other hand, incomplete. This was not unexpected, since these heteroantisera contain antibodies to a broader spectrum of Gross virus-associated antigens than mouse antisera (Herberman, 1972), some of which represent antigenic specificities (*e.g.* the $G_{IX}$ alloantigen) hitherto undetected on non-lymphoid cells (Stockert, Old and Boyse, 1971). The absorption data do not provide conclusive proof of absence of " MSV-non-MLV " antigens on FBJ sarcoma cells since MLV-FBJ determined antigens may overshadow MSV-specific components which may be present on these cells. In analogous studies neither Fefer *et al.* (1967) nor Chuat *et al.* (1969) could positively identify " MSV-non-MLV " antigens on cells transformed by MSV-M and MSV-H respectively, and more recently, lack of distinctive antigen on cells transformed by MSV has been demonstrated using an S + L − (sarcoma positive, leukaemia negative) isolate selected from among semisolid agar colonies of MSV-transformed Swiss/3T3 cells (Strouk *et al.*, 1972).

Experience in the serological analysis of cell surface antigens of neoplasms induced by murine leukaemia virus (Herberman, 1972) suggests that in the MSV-FBJ system we are likely to be dealing with a complex of virus-associated antigens, comprising a number of cellular and virion determinants, which may be further analysed by the application of more sensitive techniques such as immuno-electron microscopy (Aoki, Stephenson and Aaronson, 1973). Until the function of each antigen has been determined, no statement may be made about which, if any, of the antigens detected by different *in vitro* techniques mediates *in vivo* tumour rejection. Neoplastic transformation by oncogenic viruses appears to be associated in general with the appearance of several neoantigens. Thus, tumours induced by mammary tumour virus possesses individually specific, as well as virus-specified common antigens (Vaage, 1968; Morton, Goldman and Wood, 1969); while in the SV40 system, several apparently distinct antigens have been defined including a specific transplantation antigen (Habel and Eddy, 1963), transplantation and cell surface antigens cross-reactive with embryonic or egg antigens (Coggin, Ambrose and Anderson, 1970; Baranska, Koldovsky and Koprowski, 1970), the S antigen detected by immunofluorescence (Trevethia, Katz and Rapp, 1965) and a tumour-specific cell surface antigen detected by the isotopic antiglobulin technique (Ting and Herberman, 1971). Viral genetic material may be responsible for the appearance of some of these antigens while derepression may account for others.

This work was supported by grants from the Cancer Research Campaign and the Medical Research Council.

We thank Mr N. W. Nisbet for his support and Miss Heulwen Jones for secretarial assistance.

REFERENCES

AOKI, T., BOYSE, E. A. & OLD, L. J. (1966) Occurrence of Natural Antibody to the Gross (G) Leukaemia Antigen in Mice. *Cancer Res.*, **26**, 1415.

AOKI, T., BOYSE, E. A., OLD, L. J., DE HARVEN, E., HAMMERLING, U. & WOOD, H. A. (1970) G (Gross) and H-2 Cell-Surface Antigens: Location on Gross Leukaemia Cells by Electron Microscopy with Visually Labelled Antibody. *Proc. natn. Acad. Sci., U.S.A.*, **65**, 569.

AOKI, T., STEPHENSON, J. R. & AARONSON, S. A. (1973) Demonstration of a Cell-surface Antigen Associated with Murine Sarcoma Virus by Immuno-electron Microscopy. *Proc. natn. Acad. Sci., U.S.A.*, **70**, 742.

BARANSKA, W. P., KOLDOVSKY, P. & KOPROWSKI, H. (1970) Antigenic Study of Unfertilized Mouse Eggs: Cross Reactivity with SV40-induced Antigens. *Proc. natn. Acad. Sci. U.S.A.*, **67**, 193.

BISKIS, B. O. & FINKEL, M. P. (1969) Electron Microscopy of the FBJ Osteosarcoma Virus. In *27th Annual Proceedings of the Electron Microscopy Society of America.* Ed. C. J. Arceneaux. p. 384.

CHUAT, J. C., BERMAN, L., GUNVEN, P. & KLEIN, E. (1969) Studies on Murine Sarcoma Virus: Antigenic Characterization of Murine-sarcoma Virus Induced Tumor Cells. *Int. J. Cancer*, **4**, 465.

COGGIN, J. H., AMBROSE, K. R. & ANDERSON, N. G. (1970) Fetal Antigen Capable of Inducing Transplantation Immunity against SV40 Hamster Tumor Cells. *J. Immun.*, **105**, 524.

FEFER, A., MCCOY, J. L. & GLYNN, J. P. (1967) Antigenicity of a Virus-induced Murine Sarcoma (Moloney). *Cancer Res.*, **27**, 962.

FINKEL, M. P., BISKIS, B. O. & JINKINS, P. B. (1966) Virus Induction of Osteosarcomas in Mice. *Science, N.Y.*, **151**, 698.

HABEL, K. & EDDY, B. E. (1963) Specificity of Resistance to Tumour Challenge of Polyoma and SV40 Virus-immune Hamsters. *Proc. Soc. exp. Biol. Med.*, **113**, 1.

HARVEY, J. J. & EAST, J. (1971) The Murine Sarcoma Virus (MSV). *Int. Rev. exp. Path.*, **10**, 265.

HERBERMAN, R. B. (1972) Serological Analysis of Cell Surface Antigens of Tumors Induced by Murine Leukaemia Virus. *J. natn. Cancer Inst.*, **48**, 265.

JONES, D. B. & MOORE, M. (1973) Tumour Associated Transplantation Antigens of Neoplasms Induced by a Naturally Occurring Murine Sarcoma Virus (FBJ-MSV). *Br. J. Cancer*, **27**, 415.

JONES, D. B. & MOORE, M. (1974) Antigens of Tumours Induced by Naturally Occurring Murine Sarcoma Virus (MSV-FBJ). I. Detection of Group- and Type-specific Antigens by Complement Fixation. *Br. J. Cancer*, **29**, 21.

KELLOFF, G. F., LANE, W. T., TURNER, H. C. & HUEBNER, R. J. (1969) *In vivo* Studies of the FBJ Murine Osteosarcoma Virus. *Nature, Lond.*, **223**, 379.

KLEIN, E. & KLEIN, G. (1964) Antigenic Properties of Lymphomas Induced by the Moloney Agent. *J. natn. Cancer Inst.*, **32**, 547.

KOLDOVSKY, P., TURANO, A. & FADDA, G. (1969) Specific Transplantation Resistance Against Mouse Tumour Induced by Mouse Sarcoma Virus Harvey. *Folia biol., Praha*, **15**, 224.

LAW, L. W., TING, R. C. & STANTON, M. F. (1968) Some Biologic, Immunogenic and Morphologic Effects in Mice after Infection with a Murine Sarcoma Virus. I. Biologic and Immunogenic Studies. *J. natn. Cancer Inst.*, **40**, 1101.

LEVY, J. A., HARTLEY, J. W., ROWE, W. P. & HUEBNER, R. J. (1973) Studies of FBJ Osteosarcoma Virus. I. Biologic Characteristics of the "C"-type Viruses. *J. natn. Cancer Inst.*, **51**, 525.

MCCOY, J. L., FEFER, A., MCCOY, N. T. & KIRSTEN, W. H. (1972) Immuno-biological Studies of Tumours Induced by Murine Sarcoma Virus (Kirsten). *Cancer Res.*, **32**, 343.

MOORE, M. & WILLIAMS, D. E. (1972) Studies on the Antigenicity of Radiation-induced Murine Osteosarcomata. *Br. J. Cancer*, **26**, 90.

MORTON, D. C., GOLDMAN, L. & WOOD, D. A. (1969) Acquired Immunological Tolerance and Carcinogenesis by the Mammary Tumor Virus. II. Immune Responses Influencing Growth of Spontaneous Mammary Adenocarcinomas. *J. natn. Cancer Inst.*, **42**, 321.

OLD, L. J., BOYSE, E. A. & STOCKERT, E. (1964) Typing of Mouse Leukaemias by Serological Methods. *Nature, Lond.*, **201**, 777.

OLD, L. J., BOYSE, E. A. & STOCKERT, E. (1965) The G (Gross) Leukaemia Antigen. *Cancer Res.*, **25**, 813.

PRICE, C. H. G., MOORE, M. & JONES, D. B. (1972) FBJ Virus-induced Tumours in Mice. A Histopathological Study of FBJ Virus Tumours and their Relevance to Murine and Human Osteosarcoma Arising in Bone. *Br. J. Cancer*, **26**, 15.

RHIM, J. S., HUEBNER, R. J., LANE, W. T., TURNER, H. C. & RABSTEIN, L. (1969) Neoplastic Transformation and Derivation of a Focus-forming Sarcoma Virus in Cultures of Rat Embryo Cells Infected with a Murine Osteosarcoma (FBJ) Virus. *Proc. Soc. exp. Biol. Med.*, **132**, 1091.

STOCKERT, E., OLD, L. J. & BOYSE, E. A. (1971) The $G_{IX}$ System. A Cell Surface Allo-antigen Associated with Murine Leukaemia Virus; Implications Regarding Chromosomal Integration of the Viral Genome. *J. exp. Med.*, **133**, 1334.

STROUK, V., GRUNDER, G., FENYÖ, E. M., LAMON, E., SKURZAK, H. & KLEIN, G. (1972) Lack of Distinctive Surface Antigen on Cells Transformed by Murine Sarcoma Virus. *J. exp. Med.*, **136**, 344.

TING, C.-C. & HERBERMAN, R. B. (1971) Detection of Tumor-specific Cell Surface Antigen of Simian-virus 40-induced Tumors by the Isotopic Globulin Technique. *Int. J. Cancer*, **7**, 499.

TREVETHIA, S. S., KATZ, M. & RAPP, F. (1965) New Surface Antigen in Cells Transformed by Simian Papovavirus SV40. *Proc. Soc. exp. Biol. Med.*, **119**, 896.

VAAGE, J. (1968) Non-cross-reacting Resistance to Virus Induced Mouse Mammary Tumours in Virus Infected C3H Mice. *Nature, Lond.*, **217**, 101.

WAHREN, B. (1963) Cytotoxic Assays and Other Immunologic Studies of Leukemias Induced by Friend Virus. *J. natn. Cancer Inst.*, **31**, 411.